# A nomogram predicting the prognosis of young adult patients diagnosed with hepatocellular carcinoma: A population-based analysis

**DOI:** 10.1371/journal.pone.0219654

**Published:** 2019-07-11

**Authors:** Junjie Kong, Tao Wang, Shu Shen, Zifei Zhang, Wentao Wang

**Affiliations:** Department of Liver Surgery & Liver Transplantation Center, West China Hospital of Sichuan University, Chengdu, P. R. China; VA Boston Healthcare System, Harvard Medical School (Brigham and Women's Hospital), UNITED STATES

## Abstract

**Background:**

Few studies have reported the clinical characteristics and outcomes of young adult patients diagnosed with hepatocellular carcinoma (HCC). This study aimed to formulate a nomogram to predict the prognosis of young adult HCC patients.

**Methods:**

Young adult patients diagnosed with HCC from 2004 to 2015 were screened from the Surveillance, Epidemiology, and End Results (SEER) database. Based on the multivariate analysis results, a nomogram was constructed. The concordance index (c-index) and calibration were used to assess the predictive performance of the nomogram. The clinical benefit was measured by using decision curve analysis (DCA).

**Results:**

The mean follow-up time of the patients was 25.0±34.0 months. Gender, race, AFP level, Edmondson–Steiner classification, treatment and TNM stage were selected as independent prognostic factors and integrated into the nomogram. The c-indexes of the two sets were 0.786 and 0.775, respectively. The calibration curves showed good agreement between the nomogram-predicted probability and the actual observations. Furthermore, the DCA indicated that the nomogram had positive net benefits compared with the conventional staging system.

**Conclusions:**

The nomogram could accurately predict the prognosis of young adult HCC patients.

## Introduction

Hepatocellular carcinoma (HCC) is the most common primary liver malignancy worldwide and continues to have a high incidence [1]. Generally, most HCC patients are middle-aged and elderly. In China, it was estimated that approximately 92.9% of new male HCC cases in 2015 were diagnosed at the age of 45 or older [2]. In Japan, HBV- and HCV-related HCC were diagnosed at an average age of 55.8 and 67.8 years, respectively [3]. However, although accounting for a small number of HCC cases, patients younger than 40 years of age were reported to have more advanced cancer stage, larger tumor size and a more dismal prognosis [4, 5].

To date, few studies have addressed the clinical characteristics and outcomes of HCC in patients younger than 40 years of age. Furthermore, due to the low HCC incidence, the number of cases in such studies was limited. For instance, Niederle et al [6]. reported the outcomes of an HCC patient cohort including only 25 young patients. We also constructed a prognostic model for young HCC patients after hepatectomy, which included 423 cases [7]. Despite the larger number of patients, the outcomes were still limited since it was a single center study. Consequently, a more reliable and effective model for predicting the prognosis of young adult HCC patients is needed.

The Surveillance, Epidemiology, and End Results (SEER) Program is an authoritative source of information on cancer in the United States, covering approximately 34.6% of the U.S. population [8]. The nomogram is an effective statistical tool that can precisely predict the outcomes of individual patients [9]. In this study, we downloaded data on young adult HCC patients from the SEER registry and randomly divided these patients into a primary set and validation set. A nomogram was constructed by using the primary set, and the validation set was used to validate the nomogram.

## Materials and methods

### Data collection and processing

This study was performed using SEER*Sat software (Version 8.3.5). The SEER 18 Regs Research Data (1973–2015) was used to search for patients. We searched patients between 2004 and 2015 by using the site code C22.0 and a histological diagnosis of HCC (International Classification of Diseases for Oncology, 3rd Edition [ICD-O-3] code 8170 to 8175) from the SEER database. The primary selective criteria were patients age ≥18 and ≤40, and a total of 1050 patients were screened. Afterwards, patients who did not have a clear TNM stage or who were diagnosed at autopsy or on the death certificate only were excluded. We also excluded patients who had zero days of survival or follow-up. Finally, a cohort including 808 patients was used for further analysis. This project was approved by the Ethical Committee and Institutional Review Board of West China Hospital of Sichuan University.

Clinical traits including age at diagnosis, race/ethnicity, gender, Edmondson–Steiner classification, alpha fetoprotein (AFP), marital status at diagnosis, treatment, TNM stage (6th and 7th edition), months of survival and vital status were extracted for each patient. To conduct further analysis, the screened patients were randomly grouped into a primary set (n = 404) and a validation set (n = 404).

### Statistical analysis

The chi-squared test or Fisher’s exact test was used to compare the categorical variables between the primary set and validation set, and the continuous variables were compared by using the Mann-Whitney U-test. Survival distributions were estimated by the Kaplan-Meier method and log-rank test. Univariate and multivariate survival analyses were performed using the Cox proportional hazard model to identify factors associated with survival, and the hazard ratios (HR) and their 95% confidence intervals (95% CI) were recorded.

According to the results of multivariate survival analysis, a nomogram was constructed using R software (version 3.5.1, https://www.r-project.org/). The methods of nomogram construction and internal validation were described in previous studies [10–12]. The treatment variable was grouped into the No surgery group and the Surgery group. Calibration curves were conducted to assess the predictive accuracy of the nomogram, and Harrell’s concordance index (c-index) was used to measure the performance [13]. In addition, the c-index [14, 15] was used to compare the performance of the nomogram, AJCC (6th) and AJCC (7th) staging system. To reduce the bias, the calibration was conducted by using 1000 bootstrap samples. A larger c-index indicated a more accurate prognostic prediction of the nomogram. Finally, decision curve analysis (DCA) was applied to evaluate the performance of the nomogram and compare the accuracy of prognosis prediction between the nomogram and the AJCC (6th) and AJCC (7th) staging systems [13, 16].

The statistical analysis was performed by using SPSS 22.0 (SPSS Inc., Chicago, IL, USA) and R software (Version 3.5.1). All tests were two-sided, and p<0.05 was considered statistically significant.

## Results

### Patient characteristics

From the SEER database, a total of 808 young adult patients (aged ≥18 and ≤40 years old) who were diagnosed with HCC between 2004 and 2015 and had relatively complete follow-up details were identified by screening and included in further analysis. The clinical details of the patients are shown in Table 1. The mean age at diagnosis was 32.9±6.2 years old, and 71.3% of the patients were male. Approximately half of the patients were white in race, and 54.7% cases were single at diagnosis. The AFP level was elevated in 56.9% of patients, and 35.3% of patients were grade I/II for the Edmondson–Steiner classification. Over half (55.0%) of the patients did not receive any invasive treatment. Metastasis was not common and was found in only 143 (17.7%) patients. The mean follow-up time of the patients was 28.1±35.9 months. Meanwhile, we found that there was no significant difference in age, gender, race, Edmondson–Steiner classification, AFP level, marital status, treatment, TNM stage or months of survival between the primary set and validation set.

**Table 1:** Comparison of demographics of the derivation and validation sets.

	All patients (n = 808)	Derivation set (n = 404)	Validation set (n = 404)	Chi value	P-value
Ages (years; mean±SD)	32.9±6.2	32.9±6.3	32.8±6.2		0.654
Gender (n, %)				0.097	0.756
Male	576 (71.3)	290 (71.8)	286 (70.8)		
Female	232 (28.7)	114 (28.2)	118 (29.2)		
Race/ethnicity (n, %)				1.364	0.506
White	403 (49.9)	200 (49.5)	203 (50.2)		
Black	144 (17.8)	67 (16.6)	77 (19.1)		
Other	261 (32.3)	137 (33.9)	124 (30.7)		
Edmondson–Steiner classification (n, %)				1.243	0.537
I/II	285 (35.3)	135 (33.4)	150 (37.1)		
III/IV	123 (15.2)	64 (15.8)	59 (14.6)		
Unknown	400 (49.5)	205 (50.8)	195 (48.3)		
AFP level (n, %)				1.056	0.590
Negative/normal	208 (25.7)	101 (25.0)	107 (26.5)		
Positive/elevated	460 (56.9)	237 (58.7)	223 (55.2)		
Unknown	140 (17.3)	66 (16.3)	74 (18.3)		
Marital status at diagnosis (n, %)				0.414	0.813
single	442 (54.7)	224 (55.5)	218 (54.0)		
Partner	338 (41.8)	165 (40.8)	173 (42.8)		
Unknown	28 (3.5)	15 (3.7)	13 (3.2)		
Treatment (n, %)				2.559	0.465
No surgery	444 (55.0)	232 (57.5)	212 (52.5)		
Local tumor destruction	48 (5.9)	22 (5.4)	26 (6.4)		
Surgery resection	258 (31.9)	120 (29.7)	138 (34.2)		
Liver transplantation	58 (7.2)	30 (7.4)	28 (6.9)		
T				1.275	0.735
T1	315 (39.0)	157 (38.9)	158 (39.1)		
T2	157 (19.4)	79 (19.5)	78 (19.3)		
T3	274 (33.9)	133 (32.9)	141 (34.9)		
T4	62 (7.7)	35 (8.7)	27 (6.7)		
N				0.785	0.376
N0	716 (88.6)	354 (87.6)	362 (89.6)		
N1	92 (11.4)	50 (12.4)	42 (10.4)		
M				0.212	0.645
M0	665 (82.3)	330 (81.7)	335 (82.9)		
M1	143 (17.7)	74 (18.3)	69 (17.1)		
Survival months (mean±SD)	28.1±35.9	27.9±36.5	28.3±35.3		0.763

NOTE: AFP, alpha fetoprotein; SD, standard deviation.

### Analysis of risk factors for HCC

The mean follow-up time in the primary set was 27.9±36.5 months. As shown in Table 2, in the univariate analysis, gender, race (white was chosen for reference), Edmondson–Steiner classification (I/II was chosen for reference), AFP level, treatment, T stage, N stage and M stage were associated with overall survival (OS). In the multivariate analysis, race, AFP level, Edmondson–Steiner classification, treatment, T stage, N stage and M stage remained independently related to OS (Table 2). These seven factors were regarded as independent variables associated with OS in young adult HCC patients.

**Table 2:** Univariate and multivariate analysis of overall survival for the primary set.

Variables	Univariate analysis			Multivariate analysis		
	HR	95%CI	P-value	HR	95%CI	P-value
**Age, years**	1.012	0.992–1.032	0.248			NA
**Gender**			0.035			0.795
** Female**	1 (Reference)			1 (Reference)		
** Male**	1.364	1.021–1.820	0.035	1.040	0.772–1.402	0.795
**Race**			0.024			0.016
** White**	1 (Reference)			1 (Reference)		
** Black**	1.196	0.832–1.720	0.334	0.764	0.519–1.124	0.172
** Other**	1.470	1.115–1.939	0.006	1.320	0.967–1.803	0.080
**Edmondson–Steiner classification**			<0.001			0.021
** I/II**	1 (Reference)			1 (Reference)		
** III/IV**	2.464	1.653–3.673	<0.001	1.518	0.991–2.325	0.055
** Unknown**	2.676	1.939–3.692	<0.001	1.637	1.150–2.330	0.006
**AFP level**			<0.001			<0.001
** Negative**	1 (Reference)			1 (Reference)		
** Positive**	2.744	1.928–3.905	<0.001	2.068	1.420–3.010	<0.001
** Unknown**	1.372	0.857–2.196	0.188	0.986	0.604–1.607	0.954
**Marital status at diagnosis**			0.115			NA
** Single**	1 (Reference)			1 (Reference)		
** Partner**	0.776	0.589–0.997	0.047			NA
** Unknown**	0.722	0.354–1.472	0.371			NA
**Treatment**			<0.001			<0.001
** No surgery**	1 (Reference)			1 (Reference)		
** Surgery**	4.304	3.199–5.791	<0.001	2.136	1.058–4.311	<0.001
**T**			<0.001			<0.001
** T1**	1 (Reference)			1 (Reference)		
** T2**	1.483	1.006–2.185	0.047	1.247	0.837–1.858	0.278
** T3**	3.567	2.595–4.902	<0.001	2.210	1.559–3.133	<0.001
** T4**	4.964	3.226–7.637	<0.001	3.128	2.010–4.870	<0.001
**N**			<0.001			0.033
** N0**	1 (Reference)			1 (Reference)		
** N1**	2.064	1.468–2.903	<0.001	1.576	1.039–2.392	0.033
**M**			<0.001			
** M0**	1 (Reference)			1 (Reference)		
** M1**	2.265	1.690–3.036	<0.001	1.437	1.029–2.006	0.033

NOTE: AFP, alpha fetoprotein.

### Construction of the nomogram

**Fig 1:**
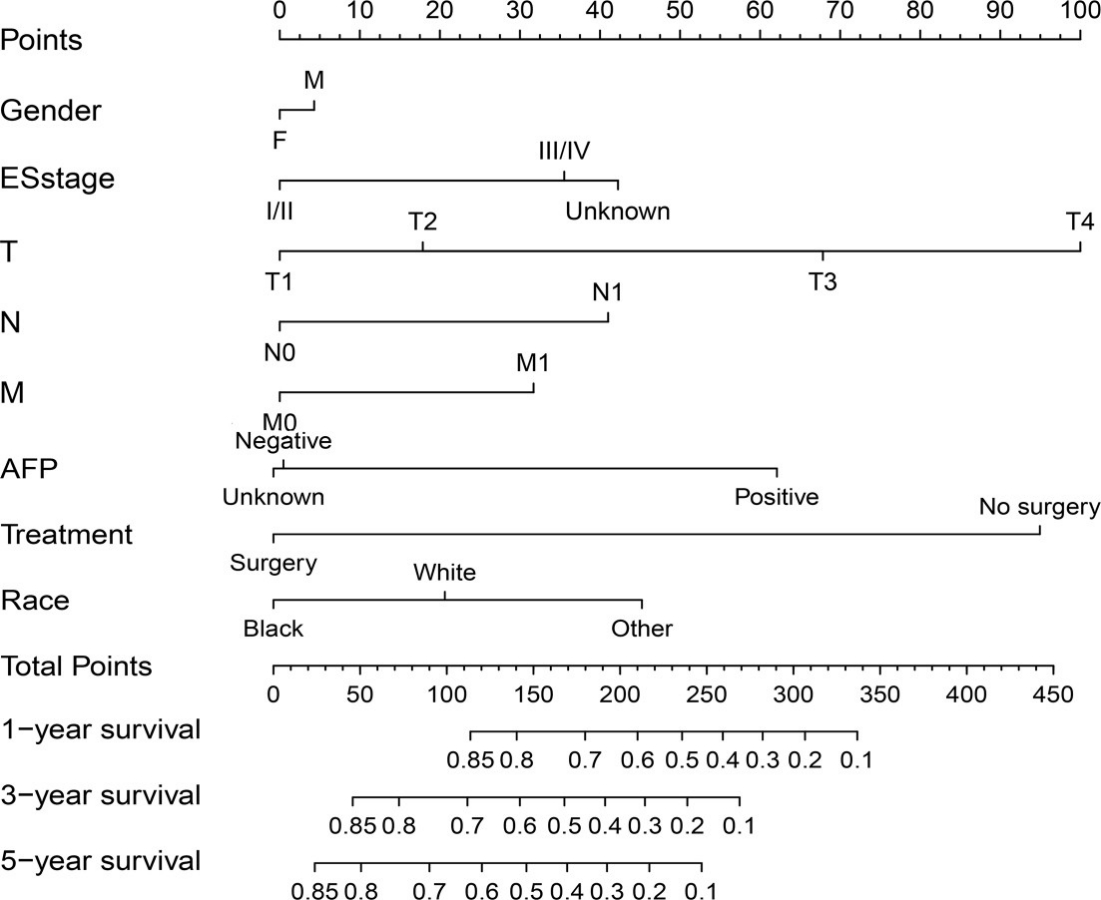
Nomogram for predicting overall survival of young adult HCC patients. For application of this nomogram, each variable axis represented an individual risk factor, and the line drawn upwards was used to determine the points of each variable. Then the total points would be calculated to obtain the probability of 1-, 3- and 5-year OS. F, female; M, male; AFP, preoperative level of serum alpha fetoprotein.

Since gender was always regarded as a predictive factor associated with HCC prognosis, this factor was integrated into the nomogram[17, 18]. In addition, taking into account the results of multivariate analysis, the following eight significant independent factors were used to generate the prognostic nomogram to predict the 1-, 3- and 5-year OS of patients: gender, race, Edmondson–Steiner classification, AFP level, treatment, T stage, N stage and M stage (Fig 1). Among the eight variables, the TNM stage, AFP level and treatment were the factors most strongly associated with the OS of young adult HCC patients.

### Validation of the nomogram

**Fig 2:**
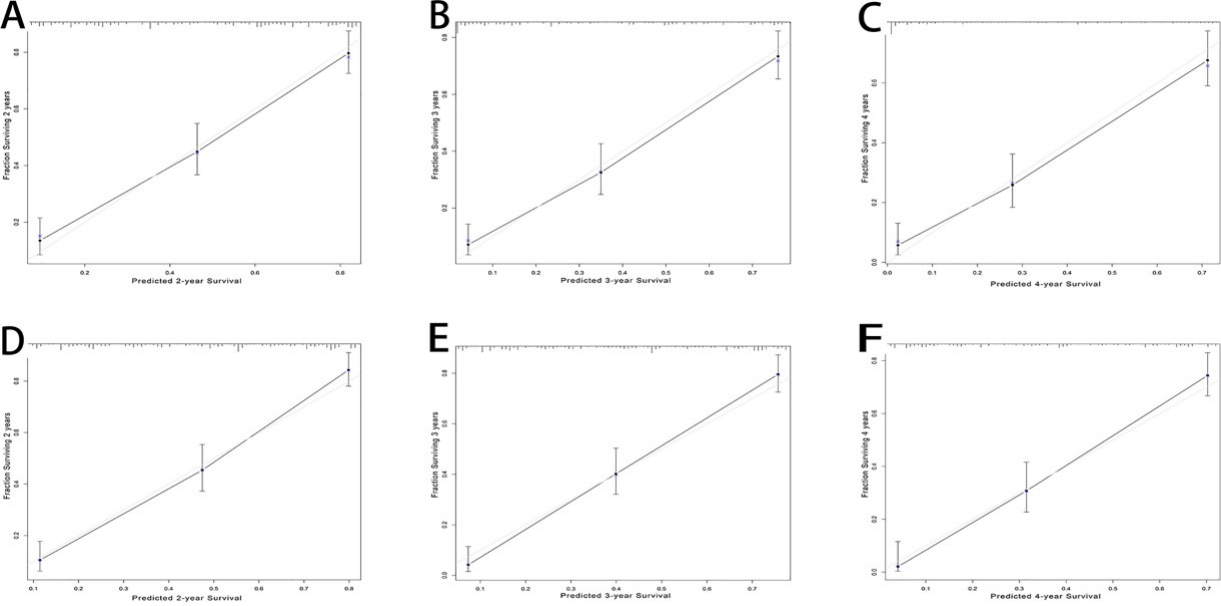
Calibration curves for the nomogram. The nomogram predicted 2- (A for primary set; D for validation set), 3- (B for primary set; E for validation set) and 4-year (C for primary set; F for validation set) overall survival. The actual 2-, 3- and 4-year overall survival was plotted on the y-axis, and x-axis showed the nomogram-predicted overall survival.

The c-index for the constructed prognostic nomogram was 0.786 (95% CI, 0.759–0.813), and the calibration curves for 2-, 3- and 4-year OS indicated good agreement between the nomogram-predicted probability and the actual observations. For the validation set, the mean follow-up time was 28.3±35.3 months. The nomogram exhibited a high accuracy of prognosis prediction (c-index = 0.775, 95% CI, 0.746–0.803) and displayed good calibration curves for the prediction of 2-, 3- and 4-year OS (Fig 2).

### Comparison of the accuracy and decision curve analysis

**Fig 3:**
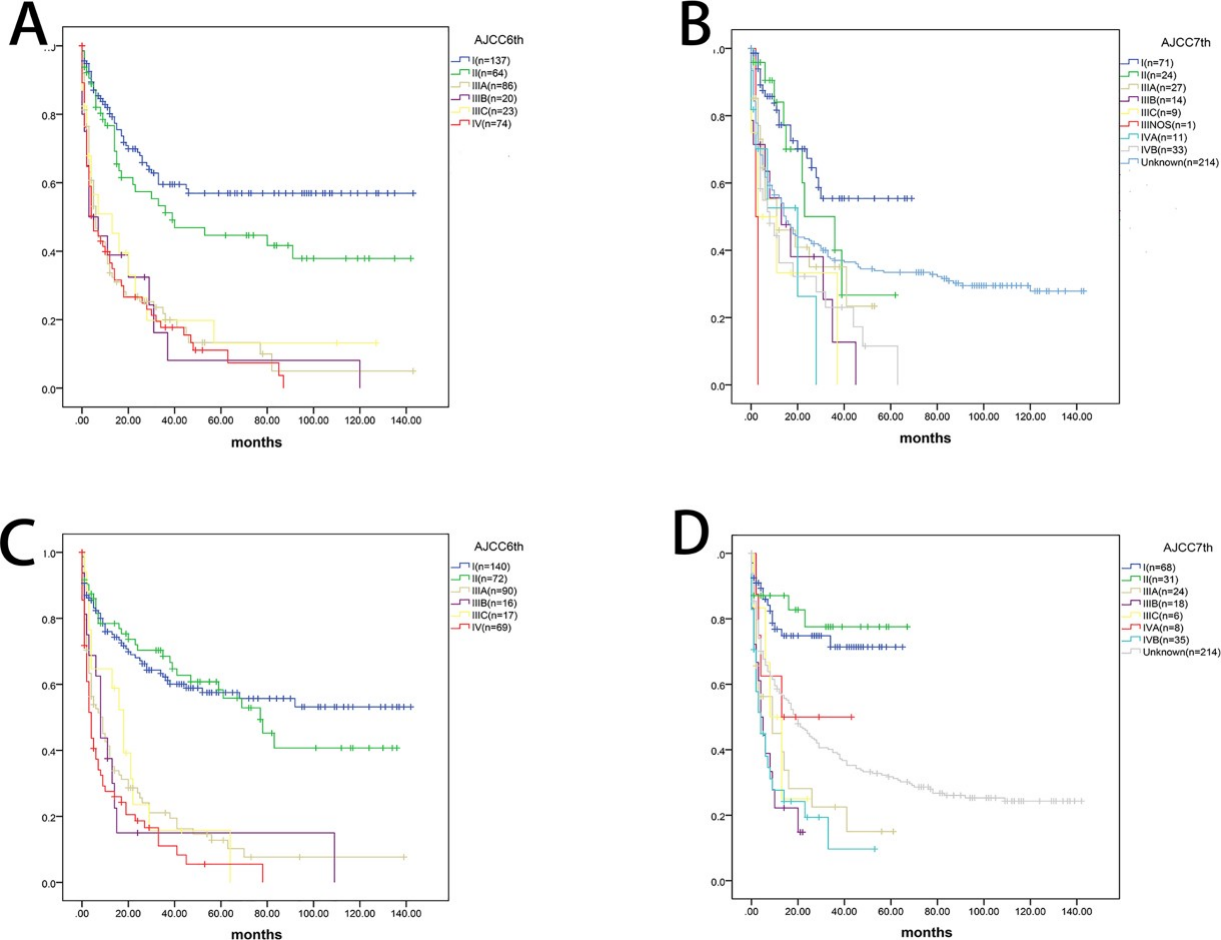
Kaplan–Meier analysis of young adult HCC patients based on AJCC (6th) and AJCC (7th) staging system. AJCC (6th) (A for primary set and C for validation set), AJCC (7th) (B for primary set and D for validation set).

**Fig 4:**
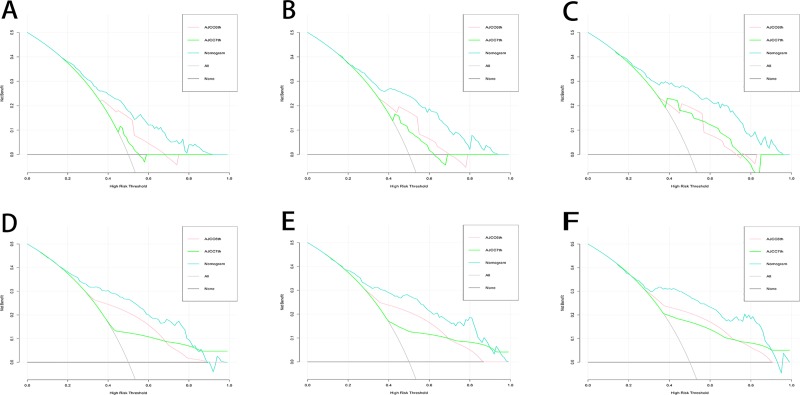
Decision curve analysis of young adult HCC patients. Decision curve analysis was used to compare the clinical net benefit between the nomogram and conventional staging systems, in terms of 2-year (A for primary set and D for validation set), 3-year (B for primary set and E for validation set) and 4-year (C for primary set and F for validation set). On decision curve analysis, the horizontal solid black line assumed no patients would die, and the solid grey line assumed all patients would die.

As shown in Fig 3, although the AJCC (6th) and AJCC (7th) staging systems had significant classification of the OS (P<0.001), the c-index (6th edition, 0.673, 95% CI 0.639–0.707; 7th edition, 0.556, 95% CI 0.521–0.591) was significantly lower than that of our nomogram (p<0.001). In the validation set, our nomogram also had a higher c-index (p<0.001) than the conventional staging systems (6th edition, 0.666, 95% CI 0.630–0.702; 7th edition, 0.547, 95% CI 0.510–0.584). Additionally, in the DCA, we found that our nomogram showed a superior net benefit across a wider scale of threshold probabilities for predicting 2-, 3- and 4-year OS than the AJCC (6th) and AJCC (7th) staging systems (Fig 4).

## Discussion

This study collected clinical characteristics of young adult HCC patients diagnosed between 2004 and 2015 from the SEER database. After screening, 808 HCC patients were finally included in the analysis and randomly separated into two groups (primary set and validation set). Subsequently, we constructed a nomogram that contained the risk factors associated with the prognosis of HCC to predict the OS of young adult HCC patients. The calibration cures for 2-, 3- and 4-year OS closely matched the ideal 45-degree line, and the c-index of the nomogram was 0.786, which was significantly higher than that of the conventional staging systems–AJCC (6th) and AJCC (7th). Furthermore, the DCA analysis also indicated that compared with the conventional staging system, our nomogram had a superior net benefit with a wider scale of threshold probabilities in the primary set and validation set.

Although HCC is one of the most common malignancies in the world and accounts for numerous cancer-related deaths, few studies have thoroughly discussed the clinical characteristics and prognosis of young adult HCC patients [2, 6, 19]. Moreover, the published studies drew different conclusions about the prognosis of young adult HCC patients. Niederle et al [6]. demonstrated that compared to the elderly patients, young adult HCC patients had a significantly higher AFP level, a lower bilirubin level and a better OS. Ha et al [20]. obtained similar results and proved that young adult HCC patients had a higher Edmondson–Steiner grade, larger tumor size and more intrahepatic metastasis. Lee et al [21]. suggested that young adults did not enjoy survival benefits in comparison with elderly HCC patients, which was consistent with Takeishi’s study [3]. Meanwhile, Takeishi et al [3]. also reported that young adult patients were more likely to experience an advanced stage in the progression of HCC. In this study, approximately three-quarters of patients (71.3%) were male, and approximately three-quarters (68.9%) of patients with known AFP levels had an elevated AFP value. Moreover, 11.4% and 17.7% had lymph node metastasis and distant metastasis, respectively. More than one half (55.0%) of the patients did not receive surgical treatment, and the reasons for the lack of cancer-related surgery were that it was not recommended or was contraindicated, which indicated that young adult HCC patients were more likely to have an end stage of cancer.

On the basis of the multivariate analysis results and taking into account the results of previous studies, gender, race, AFP level, Edmondson–Steiner classification, treatment, and TNM stage were regarded as risk factors associated with the prognosis of young adult HCC patients and were integrated into the nomogram. Among them, the TNM stage, AFP level and treatment were the variables most strongly related to the OS of young adult HCC patients. The serum AFP level was the most common laboratory value for HCC diagnosis and was used for decades [22]. The AFP level has still been considered an effective serum marker for the screening of HCC in recent years, especially in poor and remote areas [23]. We also demonstrated that the AFP value was closely related to HCC differentiation, tumor size and vascular invasion in a previous study [24]. The T stage (tumor size) was always deemed a crucial prognostic factor for HCC [25, 26] and was widely included in various conventional HCC staging systems for guiding treatment, such as the AJCC stage, BCLC stage, and Okuda stage [27]. In these stage systems, a larger tumor size usually represents a more advanced tumor stage. Malignancies with metastasis (including lymph node metastasis and distant metastasis) were considered to have progressed into an advanced stage. HCC with metastasis was always regarded as a contraindication for patients to receive surgical treatment, liver transplantation or even local tumor destruction, and the patients thus had a poor prognosis [28]. Although sorafenib has been demonstrated to be effective for prolonging the lives of these patients, the results were dismal [28, 29]. Undoubtedly, the management of HCC with metastasis remains a challenge and needs further attention.

It is not surprising that young adult HCC patients who received surgical treatment had a better OS, especially in our study. First, according to the description in the SEER database, the reasons that patients did not receive surgery were that it was contraindicated or not recommended, which indicated that these patients usually had a later stage of cancer or a worse physical condition. Consequently, these patients undoubtedly had a dismal prognosis. In contrast, Mao et al [30] demonstrated that primary tumor resection could bring survival benefit to selected HCC patients with resectable extrahepatic metastasis, which was similar to the results of Hu’s study [31]. In the future, the treatment of HCC patients with late cancer stage should be further explored, as a better treatment strategy is still needed.

Another important finding was the relatively low transplantation rate in young adult HCC patients. In this study, only 7.2% of patients received liver transplantation. Furthermore, using data obtained from the United Network of Organ Sharing (UNOS) and Organ Procurement and Transplantation Network (OPTN) databases, a recent population-based study discovered that only 464 young adult HCC patients received liver transplantation between 1987 and 2012 [32]. We speculated that the main reason was the late stage at diagnosis for young adult HCC patients. It was previously reported that young adult HCC patients usually had a higher AFP level, larger tumor size and more frequent metastasis at diagnosis [33]. Similar clinical characteristics of such patients were also discovered in this study; 61% of patients had T2 stage cancer. A later tumor stage could fail to satisfy the criteria used to select candidates for transplantation, such as the Milan criteria [34] and Hangzhou criteria [35]; as a result, very few young adult HCC patients could meet the criteria and benefit from transplantation. In addition, previous studies also proved that young adult HCC patients usually had more invasive tumor characteristics, the stage of the tumor could progress rapidly, and many patients might lose their chances to receive transplantation while on the waitlist [20, 32, 36]. However, compared with older (age >40 years) HCC patients, young adult HCC patients had a better OS after transplantation, although this difference was not significant [32]. Consequently, liver transplantation could provide good outcomes for young adult HCC patients who met the criteria for transplantation, and further exploration should be performed.

This study represented one of the largest cohorts focusing on the prognosis of young adult HCC patients. The data were collected from multiple centers, and heterogeneity in various centers could be successfully resolved. However, limitations still exist. First, this work was a retrospective study. Second, due to the limitations of the SEER database, several prognostic factors, such as microvascular invasion (MVI), hepatic virus infection, liver function and Eastern Cooperative Oncology Group performance status (ECOG-PS), were not available in this study. The presence of MVI was proven to be associated with early recurrence and poor OS; it reflects the invasiveness of HCC and is integrated into several staging systems to predict prognosis and guideline treatment [37, 38]. In addition, young adult HCC patients were discovered to have better underlying liver function and performance status than older patients [36], and these two prognostic factors could determine the treatment strategies that patients could tolerate and were related to patients’ prognosis [1]. In the BCLC staging system, ECOG-PS was regarded as an independent factor for the selection of therapeutic strategies and the prediction of prognosis. Hepatic virus infection could cause early development of HCC [39], and young adult HCC patients were also found to have a higher hepatic virus infection rate [20]. HBV infection was demonstrated to be associated with a dismal prognosis of HCC patients [40]. Although our nomogram did not integrate all the prognostic factors mentioned above, it still achieved relatively specific prediction of the prognosis of young adult HCC patients and had a significantly higher c-index than the conventional staging systems.

In conclusion, we demonstrated that gender, race, Edmondson–Steiner classification, AFP level, treatment and TNM stage were independent prognostic factors for the prognosis of young adult HCC patients. Afterwards, a reliable nomogram was constructed that could predict the prognosis of these patients. Further exploration involving more patients and more independent prognostic factors should be performed, and a nomogram with higher accuracy and specificity than ours might be constructed in the future.

## Supporting information

S1 File Young adult HCC patients. This file provided all of the information of young adult patients diagnosed with hepatocellular carcinoma in this study.
(XLSX)
